# MTUS1/ATIP3a down-regulation is associated with enhanced migration, invasion and poor prognosis in salivary adenoid cystic carcinoma

**DOI:** 10.1186/s12885-015-1209-x

**Published:** 2015-03-31

**Authors:** Tingting Zhao, Xueqiang Ding, Boyang Chang, Xiaofeng Zhou, Anxun Wang

**Affiliations:** 1 Department of Oral and Maxillofacial Surgery, the First Affiliated Hospital, Sun Yat-Sen University, 58 Zhongshan Road II, Guangzhou, Guangdong 510080 PR China; 2 State Key Laboratory of Oncology in Southern China, Sun Yat-sen University Cancer Center, Guangzhou, Guangdong 510060 PR China; 3 Center for Molecular Biology of Oral Diseases, College of Dentistry, University of Illinois at Chicago, Chicago, IL 60612 USA; 4 Department of Periodontics, College of Dentistry, University of Illinois at Chicago, Chicago, IL 60612 USA

**Keywords:** MTUS1, ATIP, Salivary adenoid cystic carcinoma, Migration, Invasion

## Abstract

**Background:**

Microtubule-associated tumor suppressor gene (MTUS1) has been identified as tumor suppressor gene in many malignant tumors. In this study, we investigated the role of MTUS1 in the development of salivary adenoid cystic carcinoma (SACC) and its functional effect on the migration and invasion of SACC.

**Methods:**

Archival clinical samples including 49 primary SACC were examined for MTUS1 expression by immunohistochemistry. Statistical analyses were performed to evaluate the correlation between MTUS1 with histopathological features and survival. The expression of MTUS1/ATIP (AT2 receptor-interacting protein) isoforms was determined in SACC tissue samples and cell lines using quantitative RT-PCR assays. Then we investigated whether the migration and invasion of SACC were mediated by MTUS1/ATIP3a using in vitro cell migration and invasion assay.

**Results:**

We confirmed that the down-regulation of MTUS1 was a frequent event in SACC, and was correlated with distant metastasis and associated with reduced overall survival and disease free survival. Isoform specific quantitative RT-PCR assays revealed that ATIP1, ATIP3a and ATIP3b were the major isoforms of the MTUS1 gene products in SACC, and were significant down-regulation in SACC as compared to matching normal tissues. For functional analyses, we found that SACC-LM cells (SACC cell line with higher migration and invasion ability) possessed a lower expression level of ATIP3a compared to SACC-83 cells (lower migration and invasion ability). Restoration of ATIP3a expression in SACC-LM cells induced anti-proliferative activity and inhibited the migration and invasion ability. Knockdown of ATIP3a promoted the proliferation, migration and invasion ability of SACC-83 cells. Restoration of ATIP3a inhibited the phosphorylation of ERK (extracellular-regulated kinase) 1/2, the expression of Slug and Vimentin in SACC-LM cells, while knockdown of ATIP3a increased the phosphorylation of ERK1/2, the expression of Slug and Vimentin in SACC-83 cells.

**Conclusions:**

Our studies confirm that MTUS1 plays an important role in the progression of SACC, and may serve as a biomarker or therapeutic target for patients with SACC. MTUS1/ATIP3a down-regulation contributes to the proliferation, migration and the invasion abilities of SACC.

**Electronic supplementary material:**

The online version of this article (doi:10.1186/s12885-015-1209-x) contains supplementary material, which is available to authorized users.

## Background

Salivary adenoid cystic carcinoma (SACC) has unique characteristics, such as perineural and perivascular invasion and distant metastasis [1]. Clinical investigations have shown that SACC has a higher incidence of distant metastasis at an early stage, ranging from 35% to 50% [2]. To date, several studies have attempted to uncover the molecular mechanism underlying the distinctive biological behaviors of SACC. A number of growth factors, including vascular endothelial growth factor (VEGF), nerve growth factor (NGF) and epidermal growth factor (EGF) have been found to stimulate invasion of SACC cells [3,4]. Other signaling molecules have also been implicated in the metastasis of SACC, including mitogen-activated protein kinase 1/2 (MAPK1/2) and snail homolog 2 (Snail2, a.k.a., Slug) [5,6]. Although substantial progress has been made in defining the genes that contribute to the initiation and progression of SACC, the mechanistic rationale for the increased metastatic capacity of SACC is still ambiguous and needs to be further investigated.

MTUS1 is identified as an 8p22 candidate tumor suppressor gene encoding a family of angiotensin II (AT2) receptor-interacting proteins (ATIP) [7]. Alternative exon utilization of this gene leads to 5 known transcript variants that code for 5 different protein isoforms of ATIP (ATIP1, ATIP2, ATIP3a, ATIP3b and ATIP4) [8,9]. The ATIP polypeptides exhibit distinct motifs in the amino-terminal portion for localization to the cytosol, nucleus or cell membrane (cytosol, nucleus and plasma membrane for ATIP1, ATIP3 and ATIP4, respectively). The down-regulation of the MTUS1 gene has been documented in many cancer types [10-20]; among the 5 isoforms, ATIP1 and ATIP3a/b have been found to exhibit tumor suppressor function [11,12]. Our recent studies also suggest that the down-regulation of MTUS1/ATIP is a frequent event in tongue squamous cell carcinoma (TSCC) and also correlated with poor differentiation and associated with reduced overall survival [21,22]. ATIP1, ATIP3a and ATIP3b are found to be the major isoforms of MTUS1 gene in tongue epithelial cells and significantly down-regulated in TSCC tissues [22]. We also find that restoration of ATIP1 expression reduced cell proliferation in TSCC cell lines [22].

To improve patient survival, a better understanding of tumor invasion and metastasis is required so that aggressive tumors can be detected early in the disease process and targeted therapeutic interventions can be developed. Although the tumor suppressor function of MTUS1/ATIP has been defined, its role in the initiation and progression of SACC has not been reported. In the present study, we aim to assess the clinical significance of MTUS1/ATIP deregulation in patients with SACC. Further we investigate the role of MTUS1/ATIP3a in the proliferation, migration and invasion ability of SACC. We found that MTUS1 down-regulation is associated with poor prognosis in SACC; MTUS1/ATIP3a down-regulation contributes to the proliferation, migration and the invasion abilities of SACC, which involved extracellular signal-regulated kinase 1/2 (ERK1/2)-Slug signaling.

## Methods

### Patient and sample

49 SACC tissues samples from patients underwent radical surgery without preoperative chemotherapy or radiotherapy and 20 normal salivary glands tissues from the surgically treated patients were collected in the Cancer Center, Sun Yat-sen University between 1998 and 2010. The clinical characteristics of normal salivary gland tissues were presented in Additional file 1: Table S1. The tumor staging was assessed according to UICC staging (TNM) system. The tumor classification was based on the histological grading system according to the WHO classification. Three histological grades were determined: Grade I, tumors with tubular and cribriform areas, but without solid components; Grade II, cribriform tumors that were either pure or mixed with <30% solid areas; and Grade III, tumors with >30% solid patterns. Survival was calculated from diagnosis to the date of latest follow-up (or death). Median duration of follow-up was 64 months (range 12–139 months). For the use of these clinical materials, the approval from the Institute Research Ethics Committee of Cancer Center, Sun Yat-Sen University was obtained (B2013-045-01).

### Immunohistochemical staining

Immunohistochemistry (IHC) was performed on 5 mm sections of formalin-fixed, paraffin-embedded tissue samples as previous description [23]. Briefly, the paraffin section was deparaffinized with xylene and rehydrated in alcohol. Antigen retrieval was treated with boiling citrate buffer (pH 6.0) and endogenous peroxidase activity was blocked by 3% H_2_O_2_. Then the sections were staining with anti-MTUS1 antibody (Aviva, San Diego, CA, USA) for overnight at 4°C and then incubated with the MaxVision™ HRP-Polymer anti-Rabbit IHC Kit (Maixin, Fuzhou, CHA); and developed with the DAB Horseradish Peroxidase Color Development Kit (Maixin, Fuzhou, CHA) and counterstained with hematoxylin. The degree of immunostaining was scored independently by three observers according to both the proportion of positively stained tumor cells and the intensity of staining as described in our previous study [23]. The proportion of positively stained tumor cells was scored as following: 0 (no positive tumor cells), 1 (<30% positive tumor cells), 2 (30-60% positive tumor cells), and 3 (>60% positive tumor cells). The intensity of staining was graded as following: 0 (no staining); 1 (weak staining = light yellow), 2 (moderate staining = yellow brown), and 3 (strong staining = brown). Using this method of assessment, we evaluated the expression of MTUS1 by determining the staining index, which scores as 0, 1, 2, 3, 4, 6, and 9. An optimal cutoff value (median) was identified: the staining index score of > 4 was used to define tumors as high MTUS1 expression and ≤ 4 as low expression of MTUS1.

### Cell culture and plasmid transfections

Paired cell lines, SACC-83 and SACC-LM, are authentic ACC cell lines and kindly presented by Dr. Shenglin Li [24]. The STR data of SACC-83 and SACC-LM cell lines from Li *et al.* [24] was present in Additional file 2: Figure S1. The SACC-LM cell line is more aggressive than SACC-83 in terms of lung-metastatic rate [24,25]. Cells were maintained in RPMI-1640 supplemented with 10% FBS, 100 U/ml penicillin and 100 μg/ml streptomycin at 37°C in a humidified incubator with 5% CO2. Expression vector containing the coding sequence of human ATIP3a is a gift from Dr. Clara [12]. For functional analyses, the ATIP3a expression vector or empty vector (pCDNA3, Invitrogen, Carlsbad, CA, USA), gene-specific siRNA for ATIP3a and control non-targeting siRNA (Genepharma, Shanghai, CHA) were transient transfected into the appropriate cells using Lipofectamine Transfection Reagent (Invitrogen, Carlsbad, CA, USA) according to the manufacturer’s instructions. Three sequences of ATIP3a siRNA were used, and then the sequence that had the best knockdown effect was chosen. The sequences of siRNA used for transfection were shown in Additional file 3: Table S2.

### In vitro cell migration and invasion assays

Transwell assays were performed to assess cell migration and invasion ability using BD BioCoat Control Cell Culture Inserts or BD BioCoat BD MatrigelTM Invasion Chamber, respectively [26]. In brief, cells were seeded in the upper Boyden chambers of the cell culture inserts. After 24 h of incubation, cells remaining in the upper chamber (for migration) or on the upper membrane (for invasion) were carefully removed. Cells adhering to the lower membrane were stained with DAPI in the dark, imaged and counted using an inverted microscope equipped with a digital camera. Three random fields were captured at 200× magnification under microscope. The number of cells on the bottom surface was compared between groups.

### Cell proliferation assays

Proliferation was measured using an MTT assay, as previously described [22]. In brief, cells were seeded in quadruplicate in 96-well plates at a density of 5 × 10^3^ cells per well. Cell proliferation was analyzed after 24 h or 48 h by incubating the cells with 1 mg/ml MTT tetrazolium salt (Sigma). Absorbance (A) at 570 nm was measured. The cell inhibition rate was calculated as (1-A_treated_/A_control_) × 100% and cell proliferation rate was calculated as (A_treated_/A_control_-1) × 100%.

### Western blot analysis

Western blots were performed as described previously [27] using specific antibodies against E-cadtherin (E-cad), Vimentin (Vim), ERK1/2, p-ERK1/2, Slug, GFP, GAPDH (Cell Signaling Technology, Beverly, MA, USA), MTUS1 (Aviva, San Diego, CA, USA) and an Immu-Star HRP Substrate Kit (BIO-RAD, Hercules, CA, USA).

### ATIP isoform-specific real time RT-PCR

The expression of ATIP isoforms was determined in SACC cell lines (SACC-83), 3 pairs of frozen parotid adenoid cystic carcinoma samples and normal parotid tissue samples using ATIP isoform-specific real time RT-PCR assays, as previously described [22]. In brief, total RNA was isolated using the RNeasy Mini kit (Qiagen, GER). First-strand cDNA was synthesized with MLV-RT (Promega, Madison, WI, USA) using random hexamer primers (Promega, Madison, WI, USA). ATIP isoform-specific real time RT-PCR was performed using exon-specific primer pairs corresponding to 5 ATIP isoforms (ATIP1, ATIP2, ATIP3a, ATIP3b and ATIP4) [22]. All reactions were performed in triplicate. Melting curve analyses were performed to ensure the specificity of the real time RT-PCR reactions. The data analysis was performed using a modified 2^-delta delta Ct^ method, with PPIA (peptidylprolyl isomerase A) as an internal reference. The clinical material for each category (i.e., PE and PACC) was provided in Additional file 4: Table S3. The relative copy numbers of each ATIP isoform were calculated as described by Benedetto [4] and shown in Additional file 5: Table S4.

### Statistical analysis

All experiments were performed in triplicate, and the data are presented as the means ± standard deviation (SD). All statistical analyses were carried out using the Statistical Package for the Social Science (SPSS, Chicago, IL), Version 13.0. A one-way ANOVA and Student’s t-tests were used to compare the differences between groups. *χ*^2^ test was used to analyze the correlation between gene expression and the clinic pathologic characteristics. Survival curves were plotted using the Kaplan-Meier method and compared with the log-rank test. Cox regression was used for both univariate and multivariate analysis. P < 0.05 in all cases was considered statistically significant.

## Results

### MTUS1/ATIP down-regulation in the development and prognosis of SACC

**Figure 1:**
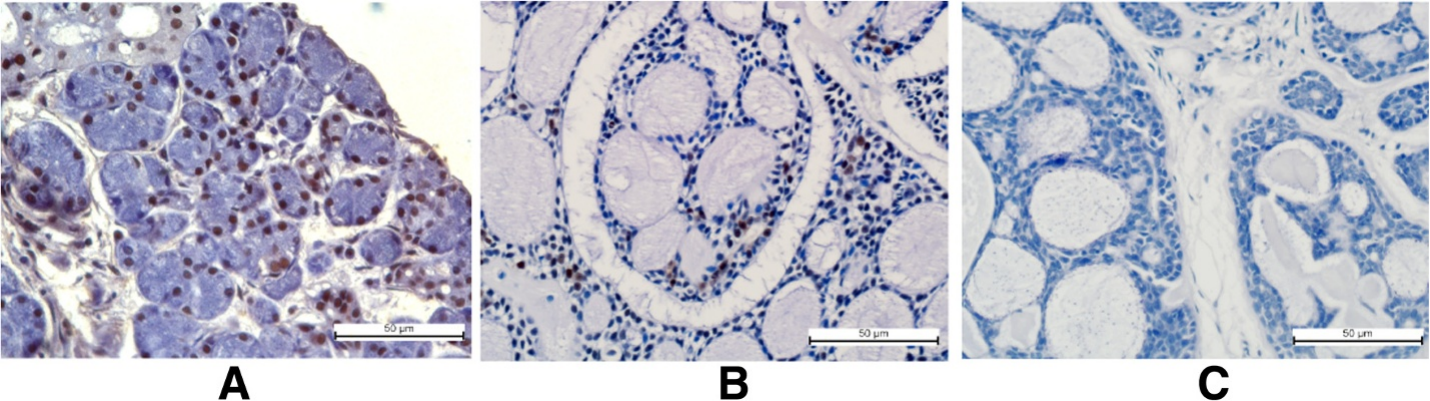
**Immunohistochemistry analyses of MTUS1/ATIP expression in normal salivary gland and SACC tissue samples.** Immunohistochemistry analyses for MTUS1/ATIP were performed as described in material and methods on **A**: normal salivary gland; **B**: salivary gland adenoid cystic carcinoma without lung metastasis; **C**: salivary gland adenoid cystic carcinoma with lung metastasis. Scale bar: 50 μm

**Figure 2:**
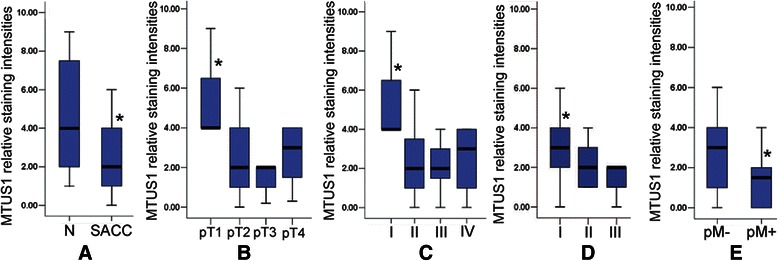
**The deregulation of MTUS1/ATIP in the development of SACC tissue.** Box plots were presented for comparing the expression of MTUS1/ATIP between normal salivary gland and SACC cases **(A)**, and in SACC cases with different tumor stages **(B)**, different clinical stages **(C)**, different histological grades **(D)**, with or without distant metastasis **(E)**. The *P*-values were computed using ANOVA and student’s T test. The boxes represent 25th to 75th percentile of the observations, and the lines in the middle of the box represent the median. *: *P* < 0.01.

To elucidate the role of MTUS1/ATIP in the development of SACC, the expression of the MTUS1/ATIP gene was examined by IHC. All the scoring of MTUS1 immunostaining was based on the figures of immunohistochemistry. As illustrated in Figure 1 and Additional file 6: Figure S2C-E, MTUS1/ATIP staining was located in both cytoplasmic and/or nuclear staining. MTUS1/ATIP was pronounced expression in normal salivary gland tissues (Figure 1A), but decreased expression in SACC samples from patients with (Figure 1B) or without (Figure 1C) lung metastasis. The MTUS1/ATIP expression was significantly decreased in primary cancer tissues compared with normal salivary gland tissues (Figure 2A). Among SACC cases, MTUS1/ATIP levels were significantly higher in pT_1_ than in other pT stages, in early clinical stage (stage I) than in the other clinical stages, in histological grade I than in other histological grades (Figure 2B-D and Additional file 6: Figure S2F-H). Statistical significant decreased MTUS1/ATIP expression was also observed in SACC samples with positive distant metastasis status (pM+) as compared to those with negative status (pM-) (Figure 2E).

The correlation between MTUS1/ATIP expression and clinicopathologic variables of patients with SACC were shown in Table 1. These observations showed that MTUS1/ATIP down-regulation was associated with pT (p =0.01), clinical stages (p = 0.0001), histological grading (p = 0.017), vital status (p =0.048), recurrence (p = 0.022), tumor site (p = 0.02) as well as the presence of distant metastasis (p = 0.005). No relationship could be found between MTUS1/ATIP expression and age (p = 0.156), and gender (p = 0.426), and pN (p = 0.957).

**Table 1:** **Correlations between MTUS1 expression and clinicopathologic characteristics of SACC patients**

		MTUS1 (cases)		
characteristics		low	High	P-value*
Gender	Male	10	8	*P* = 0.426
	Female	15	16	
Age (years)	≤46	11	15	*P =* 0.156
	>46	14	9	
Tumor stage	T_1_	0	4	*P =* 0.01
	T_2_	15	12	
	T_3_	6	0	
	T_4_	4	8	
Lymph node metastasis	Negative (pN^−^)	22	21	*P =* 0.957
	Positive (pN^+^)	3	3	
clinic stage	C1	0	4	*P =* 0.000
	C2	14	10	
	C3	5	2	
	C4	6	8	
Histological grading	I	8	15	*P =* 0.017
	II	3	5	
	III	14	4	
distant metastasis	Negtive	17	24	*P =* 0.005
	positive	8	0	
Recurrence	Negtive	16	22	*P =* 0.022
	positive	9	2	
Tumor site	Parotid	7	15	
	Submandibular	15	8	*P* = 0.02
	sublingual	3	1	
Vital status	Alive	20	21	*P* = 0.048
	Death (tumor-related)	5	3	

*: Chi-square test.

**Figure 3:**
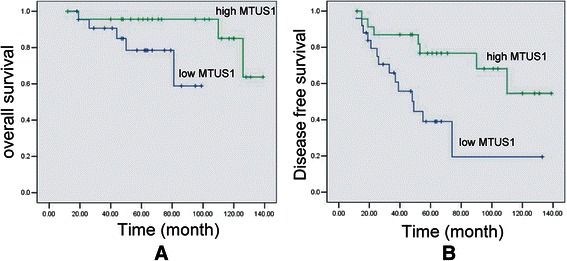
**The effects of MTUS1/ATIP expression on prognosis.** Kaplan-Meier plots of 5-years overall survival **(A)** and 5-years disease free survival **(B)** in patient groups defined by MTUS1/ATIP immunohistochemistry. The differences in survival rates are statistically significant (*P* < 0.05).

To elucidate the prognostic role of MTUS1/ATIP expression in SACC patients, we examined the relationship between MTUS1/ATIP expression and patient outcome with long-term follow-up. As illustrated in Figure 3A, a striking difference in overall survival (OS) was observed between the high MTUS1/ATIP expression group (mean survival = 84 months) and the low MTUS1/ATIP expression group (mean survival = 55 months) (p = 0.038). A statistically significant difference in survival was also observed between disease free survival (PFS) and MTUS1/ATIP expression (p = 0.008) (Figure 3B). To further evaluate the impact of MTUS1/ATIP expression and clinicopathological factors on the prognosis of SACC patients, univariate and multivariate analyses were carried out. As illustrated in Table 2, for the 5-years overall survival, both univariate and multivariate analysis indicated that MTUS1/ATIP expression, distant metastasis, histological grading and clinic stage were a significant prognostic factor for patients with SACC. But for 5-years disease free survival, both univariate and multivariate analyses indicated that only MTUS1/ATIP expression were independent prognostic factor. Thus, the above findings indicated that MTUS1/ATIP down-regulation plays an important role in the development of SACC and has a significant correlation with the poor prognosis of SACC.

**Table 2:** Univariate and multivariate analyses for 5-year overall survival and 5-year disease-free survival in SACC patients

	Univariate analysis		Multivariate analysis	
	P	Regression coefficient (SE)	P	Relative risk (95% CI)
**Overall survival**				
Clinical stage	0.02	−2.025 (1.025)	0.004	0.043 (0.005-0.367)
Expression of MTUS1	0.035	−1.653 (0.851)	0.019	29.7 (1.735-511.62)
T classification	0.038	−1.827 (1.075)	-	-
LN classification	0.733	0.376 (1.103)	-	-
Histological grading	0.033	1.344 (0.629)	0.017	5.416 (1.360-21.570)
Distant metastasis	0.002	2.498 (0.817)	0.003	26.09 (2.964-37.36)
Recurrence	0.851	0.158 (0.841)	-	-
Age	0.134	4.397 (2.934)	-	-
Gender	0.386	−0.716 (0.826)	-	-
**Disease-free survival**				
Clinical stage	0.015	−1.256 (0.517)	0.94	0.92 (0.104-8.162)
Expression of MTUS1	0.004	−1.439 (0.506)	0.004	0.255 (0.31-2.099)
T classification	0.059	−0.974 (0.516)	-	-
LN classification	0.261	−1.152 (1.025)	-	-
Histological grading	0.291	2.520 (0.239)	-	-
Distant metastasis	0.523	0.362 (0.567)	-	-
Recurrence	0.489	−0.433 (0.626)	-	-
Age	0.573	−0.250 (0.443)	-	-
Gender	0.707	−0.174 (0.464)	-	-

Note: when the *P* value of factors was less than 0.05 in the univariate analyses, the factors were then analyzed by the multivariate analyses.

### Down-regulation of MTUS1/ATIP isoforms in SACC

**Figure 4:**
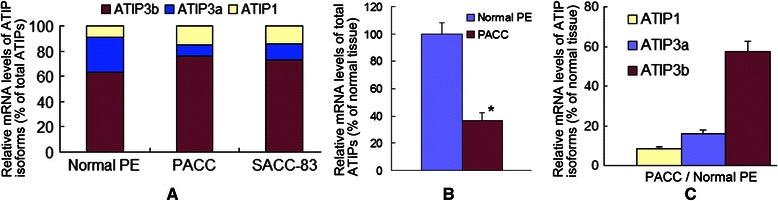
**Down-regulation of MTUS1/ATIP isoforms in SACC.** ATIP isoform-specific Real time RT-PCR assays were performed in normal parotid epithelium (PE, n = 3), parotid adenoid cystic carcinoma (PACC, n = 3) tissues and SACC-83 cells. The relative proportions of ATIP1, ATIP3a and ATIP3b transcripts are presented. **A**: Relative mRNA levels of ATIP isoforms (% of total ATIPs), **B**: Relative mRNA levels of total ATIPs (% of normal tissue), **C**: Relative mRNA levels of ATIP isoforms (% of normal tissue). Significant reductions in ATIP1, ATIP3a, ATIP3b and overall ATIP transcripts were observed in the PACC tissue in comparison to normal PE (* *P* < 0.05). ATIP2 and ATIP4 expression was minimal (less than 1%).

Our previous study had revealed that ATIP1, ATIP3a and ATIP3b are the three major isoforms of MTUS1 and are significantly decreased in TSCC [22]. In the present study, we further assessed the relative levels of each ATIP transcript variant in normal and SACC samples. As illustrated in Figure 4A, the relative proportions of ATIP1, ATIP3a and ATIP3b transcripts were 8.7%, 27.9% and 63.4% in normal parotid glandular epithelium, 15.0%, 9.2% and 75.8% in parotid ACC tissue, 14.2%, 13.2% and 72.6% in SACC-83 cell line. ATIP2 and ATIP4 expression was minimal (less than 1%).

A significant reduction in overall MTUS1/ATIP expression was confirmed in the parotid ACC tissue compared to normal parotid tissue (Figure 4B). Significant reductions in ATIP1, ATIP3a and ATIP3b were also observed in the parotid ACC tissue samples in comparison to normal parotid tissue (Figure 4C). ATIP1, ATIP3a and ATIP3b expression was decreased by 91.7%, 84.1% and 42.4%, respectively, in the parotid ACC tissue as compared with normal parotid tissue.

### MTUS1/ATIP3a overexpression is related to the inhibition of proliferation, migration and invasion in SACC

**Figure 5:**
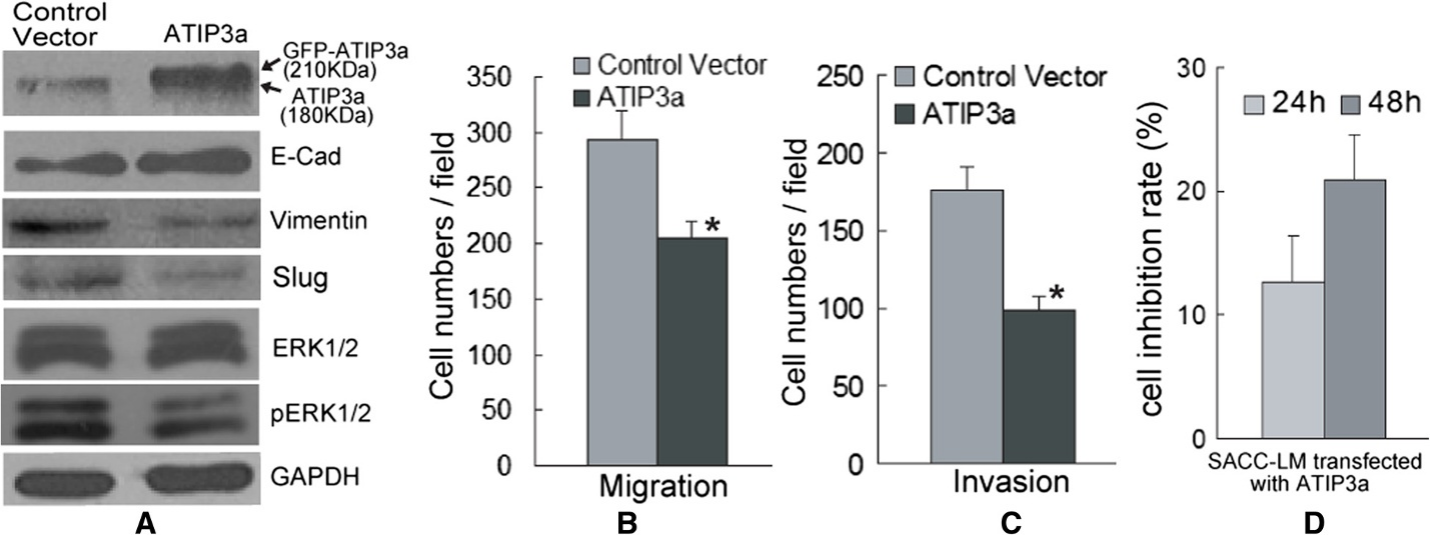
**MTUS1/ATIP3a overexpression is related to the migration and invasion of SACC. (A)** Differential expression of ERK-Slug pathway in SACC-LM cells treated with either control vector or ATIP3a plasmid. SACC-LM cells displayed decreased Slug, Vimentin and pERK1/2 protein levels and increased E-cadherin, ATIP3a protein levels upon ATIP3a overexpression. ATIP3a protein was detected by anti-MTUS1 antibody. (**B** and **C**) The migration and invasion ability of SACC cells was assessed by a transwell migration and invasion assay. ATIP3a overexpression significantly inhibited the migration **(B)** and invasion **(C)** of SACC-LM cells. **(D)** Cell proliferation was measured using a MTT assay. The cell proliferation rate of SACC-LM was significantly inhibited after overexpressed with MTUS1/ATIP3a. *: *P* < 0.05.

As shown in Additional file 7: Figure S3A, the protein level of ATIP3a in SACC-83 (lower migration and invasion abilities) was significantly higher than SACC-LM (higher migration and invasion abilities). SACC-LM cells transfected with plasmid containing MTUS1/ATIP3a cDNA (Figure 5A and S3B) displayed decreased migration and invasion abilities compared to the control plasmid transfected cells (Figure 5B and C and Additional file 8: Figure S4). We also found that several metastasis-related proteins (pERK1/2, Slug and Vimentin) were decreased and E-cadherin protein level was elevated in SACC-LM after transfected with plasmid containing MTUS1/ATIP3a cDNA (Figure 5A). Not obviously change of ERK1/2 expression was found after MTUS1/ATIP3a overexpression in SACC-LM. Moreover, SACC-LM cells transfected with plasmid containing MTUS1/ATIP3a cDNA displayed obvious morphologic changes, from irregular fibroblast-like shapes to circular or ovoid shapes (Additional file 9: Figure S5). We also found that there had significantly anti-proliferation capacity (Figure 5D) in SACC-LM cells after transfected with plasmid containing MTUS1/ATIP3a cDNA. The inhibition rate was about 21% after 48hs treatment.

### MTUS1/ATIP3a siRNA promotes proliferation, migration and invasion in SACC

**Figure 6:**
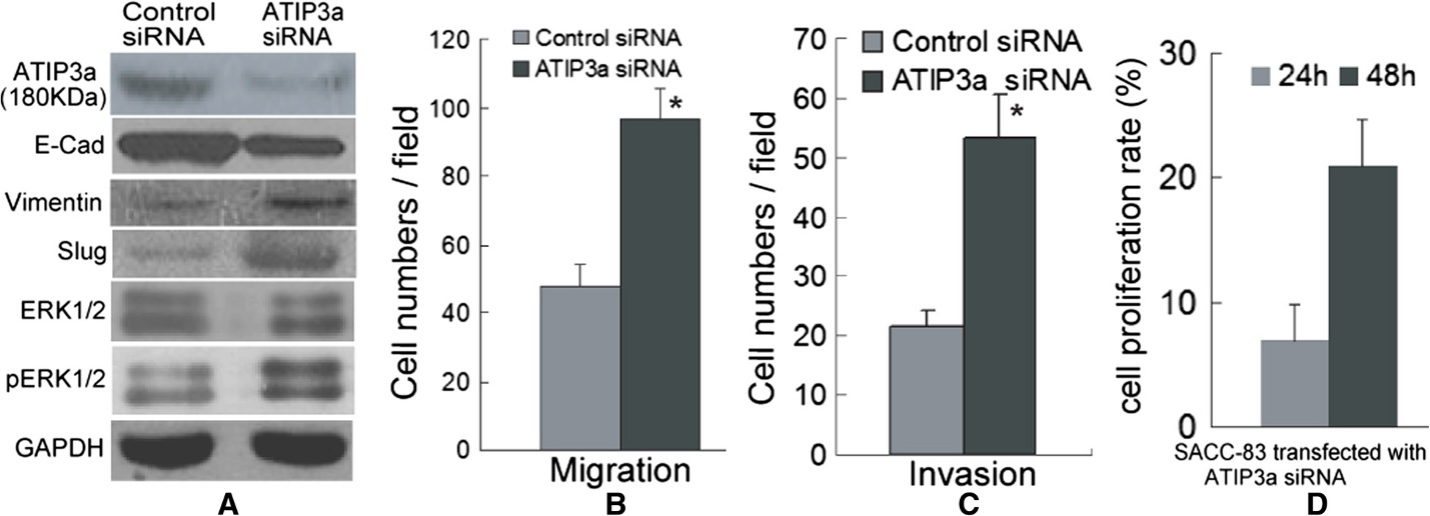
**MTUS1/ATIP3a knockdown promotes the migration and invasion of SACC. (A)** Obviously reductions in ATIP3a protein levels were observed in the ATIP3a siRNA-transfected SACC-83 cells compared to the negative control-siRNA transfected cells. SACC-83 cells displayed increased Slug, Vimentin and pERK1/2 protein levels and decreased E-cadherin protein levels upon ATIP3a knockdown. **(B, C)** ATIP3a knockdown promotes the migration and invasion of SACC-83 cells. **(D)** The cell proliferation rate of the SACC-83 cells was significantly promoted after transfection with the ATIP3a siRNA. *: *P* <0.05.

To further investigate the role of MTUS1/ATIP3a in aiding metastasis, we knocked down the expression of MTUS1/ATIP3a by RNA interference. The protein level of MTUS1/ATIP3a was significantly decreased in SACC-83 cells after transfection with the MTUS1/ATIP3a siRNA (Figure 6A). SACC-83 cells transfected with MTUS1/ATIP3a siRNA displayed increased migration and invasion abilities compared to the control siRNA transfected cells (Figure 6B and C and Additional file 8: Figure S4). After knockdown the expression of MTUS1/ATIP3a in SACC-83 cells, several metastasis-related proteins (pERK1/2, Slug and Vimentin) were significantly increased, and the protein levels of E-cadherin were significantly decreased (Figure 6A). ERK1/2 expression was not obviously change after knockdown MTUS1/ATIP3a in SACC-83 cells. Furthermore, MTUS1/ATIP3a knockdown resulted in increased cell proliferation rate (Figure 6D) in SACC-83 cells. MTUS1/ATIP3a knockdown in SACC-83 cells also induced morphologic changes consistent with EMT, with the cells displaying irregular fibroblast-like morphology (Additional file 9: Figure S5).

## Discussion

MTUS1/ATIP is one of the candidate tumor suppressor genes located in 8p22-p21.3, a region identified in our previous study as one of the most frequent LOH (87.9%) sites in oral cancer [28]. The MTUS1/ATIP gene has been detected in a large panel of normal human tissues, with the exception of bone marrow and lymphocytes, which express very little or no ATIP mRNA. ATIP3a and ATIP3b are the major ATIP transcripts in all tissues except the brain, and ATIP1 is ubiquitously expressed and ATIP4 is brain-specific [8]. Similar to Di Benedetto’s report [8] and our previous report [22], we found in the present study that ATIP1, ATIP3a and ATIP3b are the major ATIP transcripts in parotid granular tissue, whereas ATIP2 and ATIP4 are not expressed to any significant degree.

Many studies had found that the deregulation of MTUS1/ATIP is related to many types of cancer [10-15,22,29], such as hepatocellular carcinoma, bladder cancer, breast cancer, colon cancer, prostate cancer and head and neck cancer. Our recent study also found that the reduction of MTUS1 expression is associated with the development and prognosis of TSCC [21,22,28]. In the present study, we also demonstrated that down-regulation of MTUS1/ATIP is a frequent event during the progression of SACC and associates with short overall survival of the SACC patients. Thus, these findings underscore the critical contribution of MTUS1/ATIP deregulation in the tumorigenesis of SACC. We also found that the expression of MTUS1/ATIP is significantly down-regulated in parotid ACC tissue samples compared to normal parotid epithelial samples. ATIP1, ATIP3a and ATIP3b are all significantly down-regulated in these SACC tissue samples.

ATIP3a polypeptides contain a nuclear localization signal in their N-terminal portion and may not colocalize with the seven-transmembrane domain AT2 receptor inside the cell, suggesting AT2-independent roles for ATIP3 proteins in most tissues. Rodrigues-Ferreira *et al.* found that restoring ATIP3 expression in breast cancer led to reduced cancer cell proliferation, clonogenicity and anchorage-independent growth and reduced the incidence and size of xenografts grown in vivo [12]. Molina *et al.* also found that ATIP3a was related to cancer cell proliferation and metastasis [30]. In the present study, we also found that ATIP3a overexpression in SACC-LM cells significantly inhibited cancer cell proliferation, the migration and invasion ability and knockdown of ATIP3a promoted cancer cell proliferation, the migration and invasion ability in SACC-83 cells. All these results reveal that ATIP3a plays an important role in the proliferation, migration and invasion of SACC.

ERK signaling pathway had been found to play a crucial role in almost all cell functions [31]. ERK2⁄ERK1 are two isoforms of ERK that belong to the family of mitogen-activated protein kinases (MAPKs). Recent studies indicate that hundreds of proteins are under ERK-dependent control [32].

Slug belongs to the Snail family of zinc-finger transcription factors, and is a well-established downstream target of the MAPK/ERK pathway in many cell types [33,34]. Our previous study had found that MAPK-Slug pathway plays an important role in salivary adenoid cystic carcinoma (SACC) metastasis; Slug is a downstream target of MAKP1 (ERK2); siRNA-mediated ERK2-knockdown suppressed the Slug gene promoter activity and reduced the Slug protein level in SACC cells [26]. Slug gene is also best known for their roles in epithelial-mesenchymal transition (EMT) [35]. In many human cancers, there is an inverse relationship between E-cadtherin and Slug expression [34,36,37]. Wang et al. confirmed that Slug overexpression was correlated with reduced E-cadtherin expression and enhanced Vimentin expression in two independent TSCC patient cohorts. In vitro, knockdown of Slug suppressed the cell invasion and migration, in contrast, ectopic transfection of Slug led to enhanced cell invasion and migration [38]. In the present study, we found that overexpression of ATIP3a in SACC-LM cells led to an increase in E-Cadtherin and a notable decrease in pERK1/2, Slug and Vimentin. However, after knockdown the expression of ATIP3a in SACC-83 cells, the protein levels of pERK1/2, Slug, Vimentin were obviously increased, and the protein levels of E-cadtherin were obviously decreased. These results indicate that ERK1/2-Slug signaling contributes to ATIP3a induced anti-proliferation, migration and invasion in SACC.

## Conclusion

In this study, we described the expression pattern of MTUS1/ATIP in SACC for the first time. We demonstrated that down-regulation of MTUS1/ATIP plays an important role in the development of SACC and is correlated with poor prognosis. ATIP1, ATIP3a and ATIP3b are the major isoforms produced by the MTUS1 gene and are significantly decreased in SACC cells. MTUS1/ATIP3a has a notable anti-proliferation effect and inhibits the migration and invasion ability in SACC cells. Furthermore, the tumor suppressor function of ATIP3a is achieved by regulating the ERK-Slug pathway. Thus, our results provide evidence suggesting a critical role of MTUS1/ATIP in the tumorigenesis of SACC, and MTUS1/ATIP3a may serve as a biomarker or a novel therapeutic target for patients with SACC.

## Additional files

Additional file 1: Table S1. Clinical characteristics of normal salivary grand tissues.
Additional file 2: Figure S1. The STR data of SACC-83 and SACC-LM cells from Li et al [24].
Additional file 3: Table S2. The sequences of ATIP3a siRNA used for transfection.
Additional file 4: Table S3. The clinical material for each category (PE, and PACC).
Additional file 5: Table S4. The relative copy numbers of each ATIP isoform in SACC tissue and cell lines.
Additional file 6: Figure S2. Immunohistochemistry analyses of MTUS1 expression in SACC and normal salivary gland tissue samples.
Additional file 7: Figure S3. The expression level of ATIP3a protein in SACC cells was detected by western blot.
Additional file 8: Figure S4. The migration and invasion ability of SACC cells after overexpression or knockdown of ATIP3a.
Additional file 9: Figure S5. The morphology of SACC cells was detected by Immunofluorescence staining using Vimentin antibody.

## Abbreviations

MTUS1: Mitochondrial tumor suppressor 1
ATIP: AT2 receptor-interacting protein
SACC: Silivary adenoid cystic carcinoma
ERK: Extracellular-regulated kinase
MAPK1/2: Mitogen-activated protein kinase 1/2
Slug: Snail homolog 2 (Snail2)
TSCC: Tongue squamous cell carcinoma
IHC: Immunohistochemistry
