# Supplementary material for: MTUS1/ATIP3a down-regulation is associated with enhanced migration, invasion and poor prognosis in salivary adenoid cystic carcinoma
Source: BMC Cancer. 2015 Mar 31;15:203. doi: 10.1186/s12885-015-1209-x (PMC4393571; doi:10.1186/s12885-015-1209-x)
Supplement: Additional file 1: Table S1. — Clinical characteristics of normal salivary grand tissues. [file 12885_2015_1209_MOESM1_ESM.doc]

**Table S1:** **Clinical** characteristics of normal salivary grand tissues

|  |  | **No. of cases** |
| --- | --- | --- |
| **Salivary gland** | Parotid | 4 (2 from RND with TSCC and 2 from parotid benign hypertrophy ) |
|  | Submandibular | 8 ( from RND with TSCC) |
|  | Sublingual | 8 (7 from sublingual gland cyst and 1 from RND with TSCC) |

*: RND: radical neck dissection; TSCC: tongue squamous cell carcinoma
