# Supplementary material for: MTUS1/ATIP3a down-regulation is associated with enhanced migration, invasion and poor prognosis in salivary adenoid cystic carcinoma
Source: BMC Cancer. 2015 Mar 31;15:203. doi: 10.1186/s12885-015-1209-x (PMC4393571; doi:10.1186/s12885-015-1209-x)
Supplement: Additional file 2: Figure S1. — The STR data of SACC-83 and SACC-LM cells from Li et al [24]. [file 12885_2015_1209_MOESM2_ESM.doc]

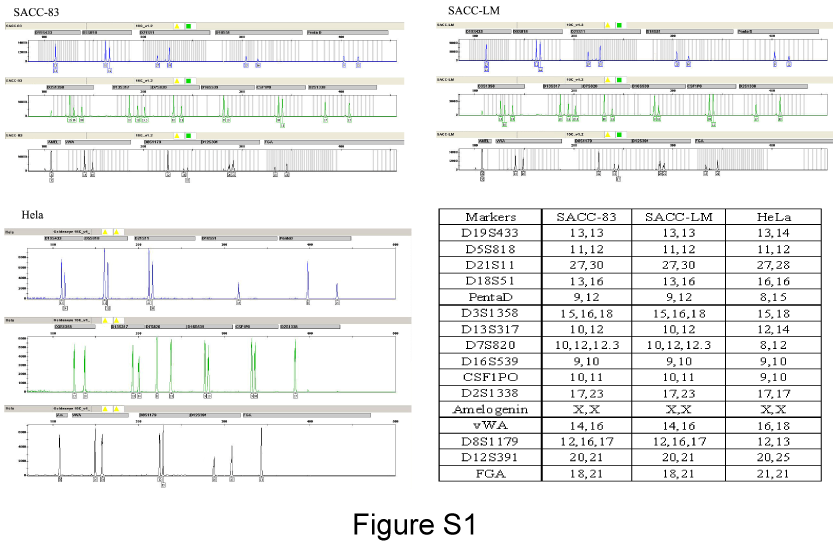


Figure S1: The STR data of SACC-83 and SACC-LM cells from Li et al [24].

STR revealed that, while DNA fingerprinting of SACC-83 and SACC-LM cells was identical, neither line was contaminated with HeLa cells. Also, the STR of SACC-83 and SACC-LM cells did not match that of any known human tumor cell lines in ATCC Profile Database.

[24] Dong L, Wang YX, Li SL, Yu GY, Gan YH, Li D, Wang CY: TGF-beta1 promotes migration and invasion of salivary adenoid cystic carcinoma. J Dent Res 2011, 90(6):804-809.
