# Supplementary material for: MTUS1/ATIP3a down-regulation is associated with enhanced migration, invasion and poor prognosis in salivary adenoid cystic carcinoma
Source: BMC Cancer. 2015 Mar 31;15:203. doi: 10.1186/s12885-015-1209-x (PMC4393571; doi:10.1186/s12885-015-1209-x)
Supplement: Additional file 3: Table S2. — The sequences of ATIP3a siRNA used for transfection. [file 12885_2015_1209_MOESM3_ESM.doc]

Table S2: The sequences of ATIP3a siRNA used for transfection

|  |  |  | Sequence |
| --- | --- | --- | --- |
| ATIP3a siRNA | No.1***** | sense | 5'-GGG UAA UCG AGG GCU UAA ATT-3' |
|  |  | antisense | 5'-UUU AAG CCC UCG AUU ACC CTT-3' |
|  | No.2 | sense | 5'-GCC CAA GAC AUG ACU UAC ATT-3' |
|  |  | antisense | 5'-UGU AAG UCA UGU CUU GGG CTT-3' |
|  | No.3 | sense | 5'-GGU GUU AGA UAU GCA UAA ATT-3' |
|  |  | antisense | 5'-UUU AUG CAU AUC UAA CAC CTT-3' |
| Control siRNA |  | sense | 5'-UUC UUC GAA CGU GUC ACG UTT-3' |
|  |  | antisense | 5'-ACG UGA CAC GUU CGG AGA ATT-3' |

*****: The sequence of No.1 was used to knockdown ATIP3a in this study.
