# Supplementary material for: MTUS1/ATIP3a down-regulation is associated with enhanced migration, invasion and poor prognosis in salivary adenoid cystic carcinoma
Source: BMC Cancer. 2015 Mar 31;15:203. doi: 10.1186/s12885-015-1209-x (PMC4393571; doi:10.1186/s12885-015-1209-x)
Supplement: Additional file 4: Table S3. — The clinical material for each category (PE, and PACC). [file 12885_2015_1209_MOESM4_ESM.doc]

Table S3: The clinical material for each category (PE, and PACC)

| Sample for PE and PACC |  |  |  |
| --- | --- | --- | --- |
| 1 | Female | 38 | parotid adenoid cystic carcinoma |
| 2 | Female | 51 | parotid adenoid cystic carcinoma |
| 3 | Male | 49 | parotid adenoid cystic carcinoma |

PE (normal parotid epithelium) and PACC (parotid adenoid cystic carcinoma) were from same patient with parotid adenoid cystic carcinoma. All the patients were underwent radical surgery and the normal epithelium was excised nearby the lesion.
