# Supplementary material for: MTUS1/ATIP3a down-regulation is associated with enhanced migration, invasion and poor prognosis in salivary adenoid cystic carcinoma
Source: BMC Cancer. 2015 Mar 31;15:203. doi: 10.1186/s12885-015-1209-x (PMC4393571; doi:10.1186/s12885-015-1209-x)
Supplement: Additional file 5: Table S4. — The relative copy numbers of each ATIP isoform in SACC tissue and cell lines. [file 12885_2015_1209_MOESM5_ESM.doc]

Table S4: The relative copy numbers of each ATIP isoform in SACC tissue and cell lines

|  | ATIP1 | ATIP3a | ATIP3b | ATIP4 | Total ATIPs |
| --- | --- | --- | --- | --- | --- |
| Normal PE | 284.7 | 1007.6 | 3273.6 | 1.0 | 4565.9 |
|  | 374.3 | 886.5 | 1779.8 | 1.0 | 3040.6 |
|  | 301.5 | 1213.8 | 2004.4 | 1.0 | 3519.7 |
| PACC | 51.5 | 20.2 | 207.9 | 1.0 | 279.7 |
|  | 22.6 | 24.4 | 152.4 | 1.0 | 199.3 |
|  | 40.5 | 25.1 | 217.2 | 1.0 | 282.8 |
| SACC-83 | 61.7 | 43.3 | 232.3 | 1.0 | 337.3 |
|  | 37.1 | 54.6 | 278.2 | 1.0 | 369.9 |
|  | 45.2 | 35.6 | 223.7 | 1.0 | 304.4 |

The copy number of ATIP4 was dedicated to 1 in the present study. The copy number of other ATIP isoform was presented as compared with ATIP4. Total ATIPs= ATIP1+ATIP3a+ATIP3b.
