# Supplementary material for: MTUS1/ATIP3a down-regulation is associated with enhanced migration, invasion and poor prognosis in salivary adenoid cystic carcinoma
Source: BMC Cancer. 2015 Mar 31;15:203. doi: 10.1186/s12885-015-1209-x (PMC4393571; doi:10.1186/s12885-015-1209-x)
Supplement: Additional file 6: Figure S2. — Immunohistochemistry analyses of MTUS1 expression in SACC and normal salivary gland tissue samples. [file 12885_2015_1209_MOESM6_ESM.doc]

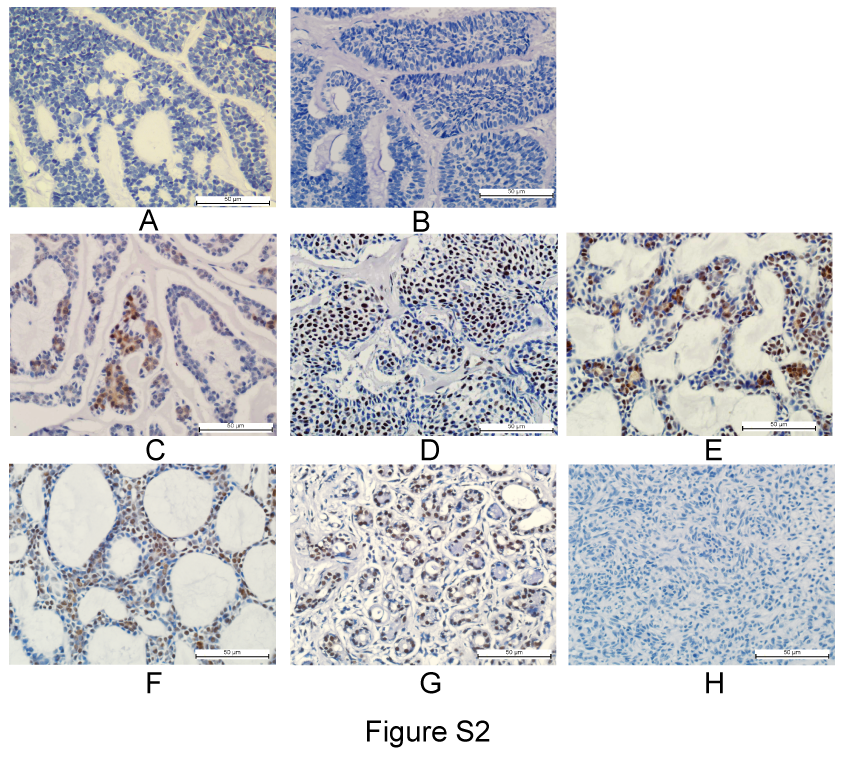


**Figure S2: Immunohistochemistry analyses of MTUS1 expression in** **SACC and normal salivary gland tissue samples**

Immunohistochemistry analyses for MTUS1 were performed as described in material and methods. A: negative control; B: Isotype control, Rabbit (DA1E) mAb IgG XP (Cell Signaling Technology) was used as isotype control; (C~E): MTUS1 expression was observed in cytoplasmic (C), nuclear (D) or both (E). (F~H): MTUS1 expression was different expression in the different histological types of adenoid cystic carcinoma, which was relatively higher expression in tubular type (F) and cribriform type (G) than in solid type (H). Scale bar: 50μm.
