# Supplementary material for: MTUS1/ATIP3a down-regulation is associated with enhanced migration, invasion and poor prognosis in salivary adenoid cystic carcinoma
Source: BMC Cancer. 2015 Mar 31;15:203. doi: 10.1186/s12885-015-1209-x (PMC4393571; doi:10.1186/s12885-015-1209-x)
Supplement: Additional file 7: Figure S3. — The expression level of ATIP3a protein in SACC cells was detected by western blot. [file 12885_2015_1209_MOESM7_ESM.doc]

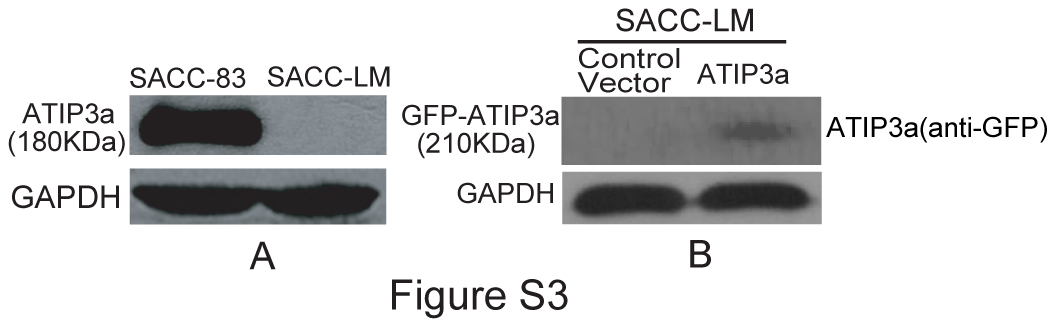


**Figure S3: The expression level of ATIP3a protein in SACC cells was detected by western blot**

(A) Significant higher expression levels of ATIP3a protein were observed in SACC-83 cells compared to SACC-LM cells (detected by antibody of MTUS1). (B) The expression levels of ATIP3a protein in SACC-LM cells after transfected with plasmid containing ATIP3a cDNA (detected by antibody of GFP).
