# Supplementary material for: MTUS1/ATIP3a down-regulation is associated with enhanced migration, invasion and poor prognosis in salivary adenoid cystic carcinoma
Source: BMC Cancer. 2015 Mar 31;15:203. doi: 10.1186/s12885-015-1209-x (PMC4393571; doi:10.1186/s12885-015-1209-x)
Supplement: Additional file 8: Figure S4. — The migration and invasion ability of SACC cells after overexpression or knockdown of ATIP3a. [file 12885_2015_1209_MOESM8_ESM.doc]

**
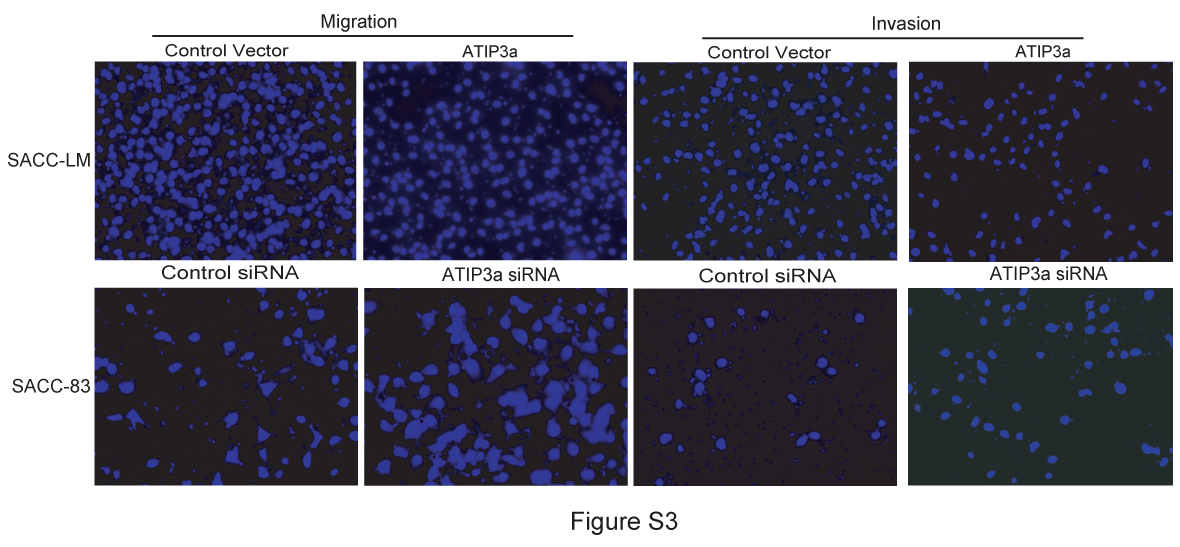
**

**Figure S4:** **The migration and invasion ability of SACC cells after overexpression or knockdown of ATIP3a.**

The migration and invasion ability of SACC cells was assessed by a transwell migration and invasion assay. ATIP3a overexpression significantly inhibited the migration and invasion of SACC-LM cells, at the same time, TIP3a knockdown inhibited the migration and invasion of SACC-LM cells.
