# Supplementary material for: MTUS1/ATIP3a down-regulation is associated with enhanced migration, invasion and poor prognosis in salivary adenoid cystic carcinoma
Source: BMC Cancer. 2015 Mar 31;15:203. doi: 10.1186/s12885-015-1209-x (PMC4393571; doi:10.1186/s12885-015-1209-x)
Supplement: Additional file 9: Figure S5. — The morphology of SACC cells was detected by Immunofluorescence staining using Vimentin antibody. [file 12885_2015_1209_MOESM9_ESM.doc]

**
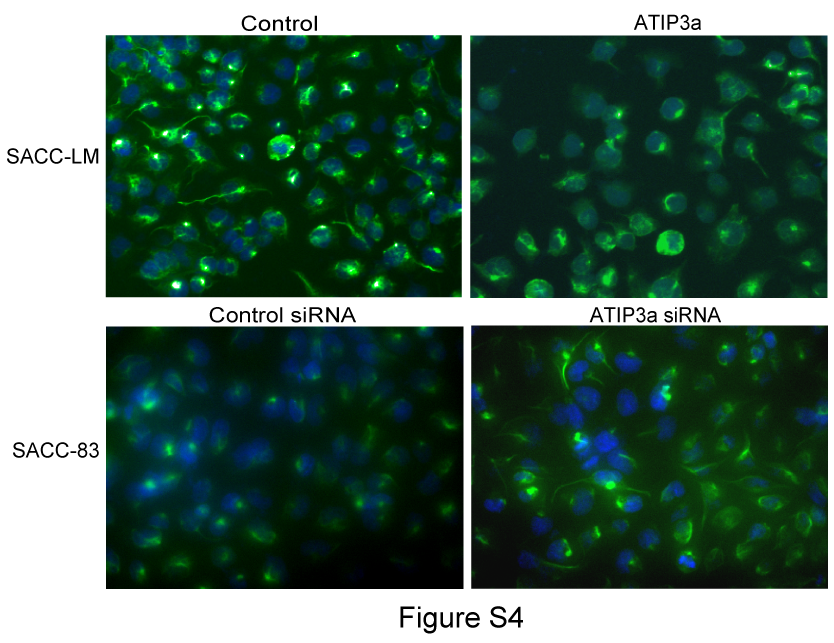
**

**Figure S5:** **The morphology of SACC cells was detected by Immunofluorescence staining using Vimentin antibody.**

SACC-LM cells transfected with plasmid containing MTUS1/ATIP3a cDNA displayed obvious morphologic changes, from irregular fibroblast-like shapes to circular or ovoid shapes, at the same time, MTUS1/ATIP3a knockdown in SACC-83 cells also induced morphologic changes consistent with EMT, with the cells displaying irregular fibroblast-like morphology. **Methods:** Immunofluorescence staining was performed as our previously described[Liu M, Wang J, Huang H, Hou J, Zhang B, Wang A. miR-181a-Twist1 pathway in the chemoresistance of tongue squamous cell carcinoma. Biochem Biophys Res Commun. 2013;441(2):364-70.]. Briefly, the cells were cultured on 22-mm coverslips in 6-well plates and then fixed in 4% paraformaldehyde. Then, the cells were incubated with primary antibodies against Vimentin (Santa Cruz, CA, USA), followed by incubation with the secondary antibody. The slides were examined using a fluorescence microscope (Olympus). The green fluorescence shows the localization of Vimentin, nuclei were counterstained with 4`,6-diamidino-2-phenylindole (DAPI). Representative images of the cells were captured at a magnification of 400 x.
